# *Drosophila* Rif1 is an essential gene and controls late developmental events by direct interaction with PP1-87B

**DOI:** 10.1038/srep10679

**Published:** 2015-05-29

**Authors:** Easwaran Sreesankar, Vellaichamy Bharathi, Rakesh K. Mishra, Krishnaveni Mishra

**Affiliations:** 1 Department of Biochemistry, School of Life Sciences, University of Hyderabad, Hyderabad- 500 046, INDIA; 2 Centre for Cellular and Molecular Biology, Uppal road, Hyderabad-500 007, INDIA

## Abstract

Rif1, identified as a regulator of telomerase in yeast, is an evolutionarily conserved protein and functions in diverse processes including telomere length regulation, epigenetic gene regulation, anti-checkpoint activity, DNA repair and establishing timing of firing at replication origins. Previously we had identified that all Rif1 homologues have PP1 interacting SILK-RVxF motif. In the present study, we show that *Drosophila* Rif1 is essential for normal fly development and loss of dRif1 impairs temporal regulation of initiation of DNA replication. In multiple tissues dRif1 colocalizes with HP1, a protein known to orchestrate timing of replication in fly. dRif1 associates with chromosomes in a mitotic stage-dependent manner coinciding with dephosphorylation of histones. Ectopic expression of dRif1 causes enlarged larval imaginal discs and early pupal lethality which is completely reversed by co-expression of PP1 87B, the major protein phosphatase in *Drosophila*, indicating genetic and functional interaction. These findings suggest that dRif1 is an adaptor for PP1 and functions by recruiting PP1 to multiple sites on the chromosome.

Rif1, Rap1 interacting factor 1, was first identified as a negative regulator of telomerase in budding yeast^1^ and subsequently in fission yeast^2^. In budding and fission yeasts, Rif1 is required to prevent the elongation of long telomeres and in addition, also influences telomere position effect^1^,^2^,^3^,^4^,^5^. Mammalian Rif1 homologues are known to be critical for DNA repair^6^,^7^,^8^. Several recent studies have implicated a role for mammalian Rif1 in the choice of repair pathway for damaged DNA. Specifically, Rif1 is required to prevent broken DNA ends from entering the homology directed pathway of DNA repair and instead, channel repair through the non-homologous end joining^9^,^10^. Choice of DNA repair pathway is critical for maintaining genome integrity; in G2 phase, the error-free homologous recombination is the preferred pathway due to the availability of the sister chromatin while in G1, non-homologous end joining is preferred. Further studies provide a plausible mechanistic basis for mammalian Rif1 function by showing that Rif1 opposes the end-resection by BRCA1 and C-terminal binding protein-interacting protein (CtIP)^11^,^12^,^13^. Earlier studies in *S. cerevisiae* had shown that Rif1 could also function like an anti-checkpoint factor and prevent the addition of telomeric repeats to double strand breaks occurring in non-telomeric regions^14^,^15^.

One function of Rif1, which is conserved in yeast and mammals, is its role in regulating the timing of firing at replication origins. In a screen for genes that affect the timing of firing at replication origins in *S. pombe*, it was found that in *rif1-*Δ, many late firing origins fired early in S phase and firing of a few early origins was delayed^16^. A similar role for mammalian Rif1 in regulating timing of replication initiation in an origin-specific manner was demonstrated^17^,^18^. Both these studies also proposed that Rif1 may influence higher order chromatin structures to modulate timing of origin firing.

While these studies highlight the varied functions of Rif1, the molecular basis of these functions was not clear. To gain more insight into the diversification of Rif1 functions we had previously carried out a detailed bioinformatic analysis and found that Rif1 homologues exist in all animals and fungi, although clear plant homologues could not be detected. We also identified a conserved PP1 interacting SILK-RVxF motif as the most conserved feature in Rif1 across evolution. Following this observation, it was demonstrated recently in yeast that Rif1 indeed interacts with PP1 and this interaction was required for regulating timing of replication^19^,^20^,^21^. Our earlier results had shown that *Drosophila* Rif1 (dRif1) expresses abundantly in the S2 cells but does not have a critical role in DNA damage repair. In order to understand how dRif1 functions in *Drosophila* in a developmental context, we initiated a detailed analysis of dRif1 protein in fly. We find that dRif1 is present in early embryos and associates with chromosomes in a dynamic manner in different mitotic stages of the cell cycle. We find that dRif1 is an essential gene and its ectopic expression leads to lethality which can be completely reversed by the simultaneous expression of the protein phosphatase, PP1-87B. Finally, we show that PP1 and dRif1 interact molecularly. Our results suggest that dRif1 may execute its function(s) by recruiting PP1 to various target loci.

## Results

### dRif1 is abundantly expressed in early embryos

**Figure 1:**
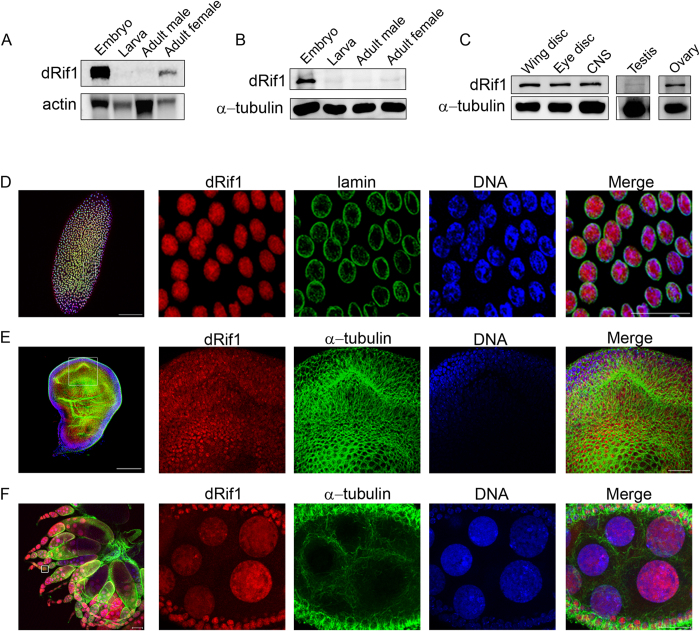
Expression and localization of dRif1 in different developmental stages. Presence of dRif1 across fly developmental stages was assessed by the presence of RNA(A) and protein(B-C). Uncropped images are shown in the supplementary figure S1. Localization of the protein across developmental stages are shown from E-F (scale bar represents 100 μm in the lower magnification image and 20 μm in the higher magnification insets). **A**) Northern blots for total RNA from indicated tissues are probed with full length dRif1 and the same blot probed with actin served as a loading control. **B**) Total protein from the indicated developmental stages were extracted and analysed by western blotting with dRif1 and the same blot probed with α-tubulin antibody serves as loading control. **C**) Protein extracts from wing imaginal disc, eye imaginal disc, CNS, adult ovary and testis were loaded separately and probed with dRif1 and α-tubulin on the same blot served as a loading control. **D**) A single embryo stained with antibodies to dRif1 (red), nuclear lamin (green) and DAPI (blue) stains the DNA. Insets show the marked magnified regions of the embryo. **E**) Localization of dRif1 (red) in the nuclei of larval wing imaginal disc. Anti-tubulin staining is in green and DAPI is blue. **F**) In the ovary dRif1 (red) localized to the follicle cells (smaller nuclei) and nurse cells (large nuclei). Tubulin staining is in green and DNA stained with TOPRO-3 is in blue.

In our earlier work, we had identified a *Drosophila* homologue of Rif1 (dRif1) and showed that it is expressed in the embryo derived S2 cell line^22^. We showed that unlike the earlier reported roles for Rif1 in other organisms, neither is dRif1 involved in telomere silencing nor does it localize to DNA double-strand breaks. To investigate the function of dRif1 in flies, we analysed dRif1 expression in the various developmental stages. Northern blotting analysis of RNA isolated from embryos, larvae and adults showed that the dRif1 mRNA is abundant in the embryonic stages and in the adult female but was negligible in larvae or adult male (Fig. 1A). To test if the presence of RNA reflects the presence of protein, western blots were performed using antibodies to dRif1. As shown in Fig. 1B and S1, the embryonic stages have dRif1 protein but the levels are extremely low in adults and undetectable in larvae. These data indicate that the protein expression is very low in later stages of development and that the mRNA in the adult female is for maternal deposition. We reasoned that it is also possible that the protein is expressed only in specific tissues in larvae and also in the female specific tissues in the adult and therefore difficult to detect in the western blot with whole animal extracts. In order to test this possibility, we dissected out a few larval imaginal discs, ovary and testis separately and loaded protein from 10 larval equivalents of eye imaginal disc, wing imaginal disc and CNS. The western blot shows that indeed dRif1 is expressed in imaginal discs, the CNS and ovary and at extremely low levels in testis (Fig. 1C). However, amount of protein present in these adult tissues is much lower than in embryos, suggesting that it has a major function early in development and in proliferating tissues later.

We followed up our expression analysis with immunostaining experiments at different developmental stages of *Drosophila*. dRif1 localizes to the nuclei of early (0-2 hr old) embryo (Fig. 1D), imaginal disc in larvae (Fig. 1E) and ovarian cells in the adult female (Fig. 1F). In the ovary, it expresses both in the nurse cells and follicle cells, implying that it is maternally deposited. Our findings suggest that, dRif1 has essential function(s) in early development; although a possible additional specific function in the ovary cannot be ruled out from these studies.

### dRif1 is present in dividing cells of the developing embryo

**Figure 2:**
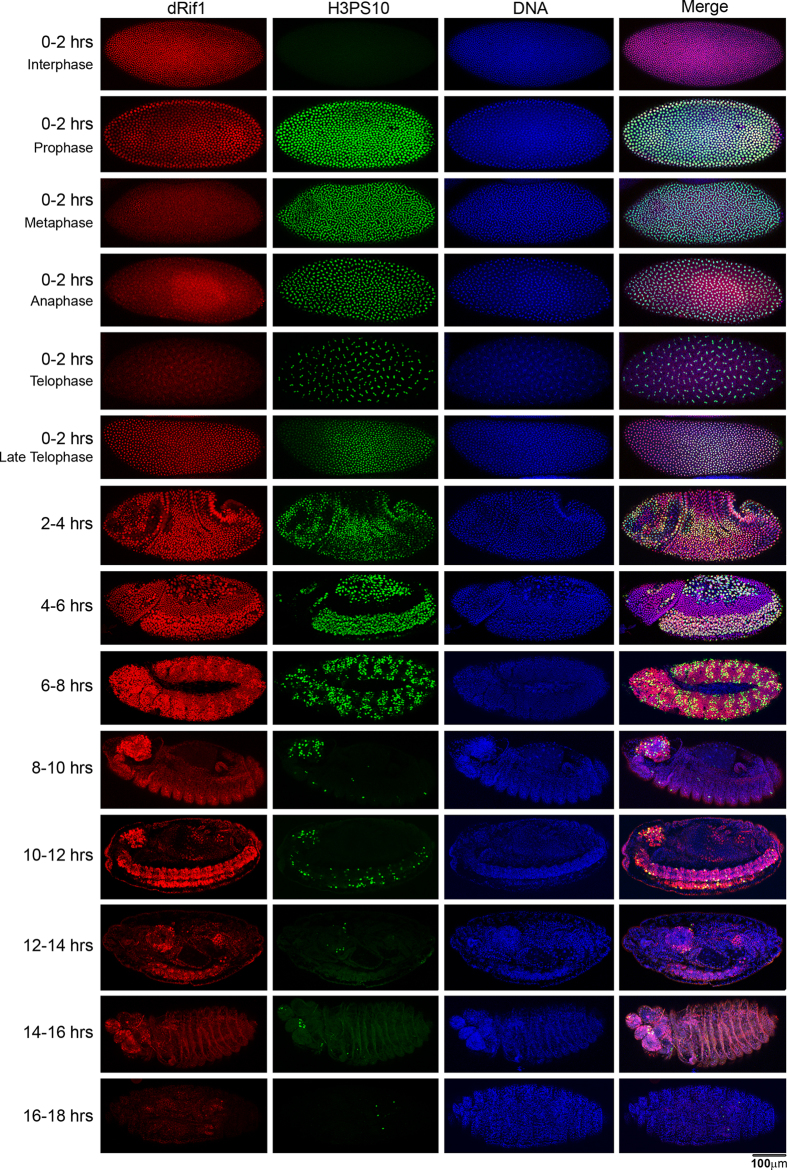
Localization of dRif1 in embryonic development. Embryos of the indicated stages of development were stained with antibodies to dRif1 and H3PS10. The first 4 rows show 0 to 2 hour synchronously dividing embryos in various stages of mitosis. Up to 4 hour of embryonic development, dRif1 (red) was found in all nuclei with similar intensity. In 4 to 8 hour of development, dRif1 was high in regions of the embryo where H3PS10 (green) also stained. In 8 to 14 hours, the embryonic CNS was intensely stained with dRif1, and as it progressed beyond 14 hours dRif1 protein was not present in any of the nuclei in all the embryos. Nuclei were stained with DAPI, which is in blue color. All the embryos were collected as 0-2 hour old and staged as labeled and stained separately. Embryos are oriented as anterior to the left and scale bar represents 100 μm.

We examined the localization of dRif1 protein through different embryonic stages. We collected embryos for 0-2 hr and incubated at 25 °C for different time intervals as specified in Fig. 2. Embryos were stained for dRif1 along with antibodies specific for histone H3 phospho-serine10 (H3PS10), marker for mitosis that identifies domains of active cell divisions. Up to 4 hr, dRif1 was localized to all nuclei almost uniformly. Following this and up to 8 hrs, a pattern begins to emerge where dRif1 is high in certain regions of the embryos coinciding with H3PS10. Thereafter, up to 14 hrs, the embryonic CNS shows intense staining for dRif1 and H3PS10 while rest of the embryo has negligible levels of the two proteins. As development progressed beyond 14 hrs, dRif1 gradually diminishes and disappears. These observations suggest that dRif1 is expressed in actively dividing embryonic cells/mitotic domains and the expression is very low in non-dividing cells.

### Cell cycle stage specific chromosomal association of dRif1

**Figure 3:**
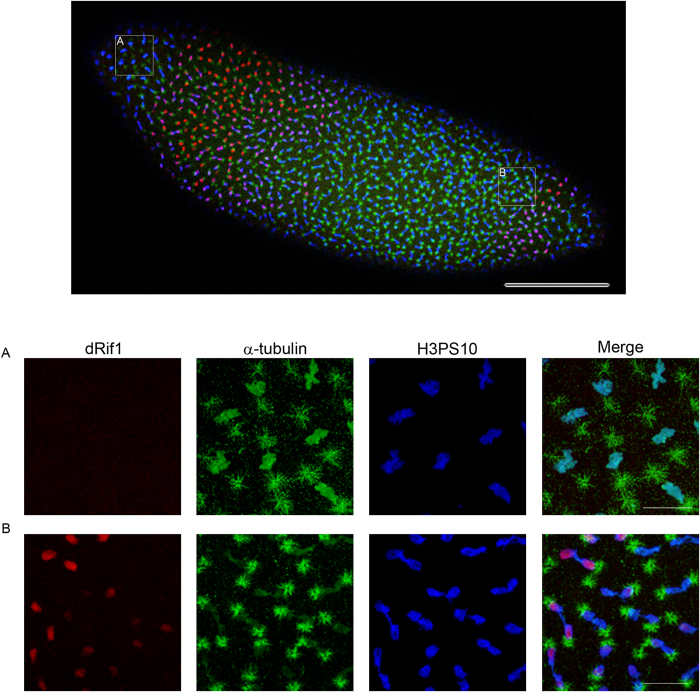
dRif1 localization across cell cycle. A single embryo with nuclei in various stages of mitosis stained with dRif1 antibody (red) along with mitotic marker-H3PS10 (blue) and tubulin (green). Higher magnification images (**A** and **B**) are from marked regions of the same embryo showing dynamic association of dRif1 on separated anaphase chromosomes. The scale bar represents 100 μm in the lower magnification image and 20 μm in the higher magnification insets.

In the localization experiments described above, we observed marked differences in the localization of dRif1 in different nuclei of early embryos and this appeared to be because of difference in cell division stages. To investigate this in more detail we immuno-localized dRif1 in conjunction with cell cycle markers to test whether the dRif1 localization oscillates in a cell cycle stage dependent manner. H3PS10 antibody was used to mark mitotic cells and PCNA was used as an early G1-S phase marker (Figure S2). We have used 0-2 hr old embryos which are in a stage before the mid-blastula transition and are undergoing synchronized mitotic cycles^23^. dRif1 could be detected in almost all interphase cells and in prophase cells while in metaphase, when the chromosomes are at the midzone, it could not be detected on the chromosomes but reappeared at the end of anaphase (Figure S2). Using H3PS10 to mark condensed chromosomes and α-tubulin as a mitotic spindle marker, we observe that dRif1 is absent on metaphase chromosomes (Fig. 3A) and associates with the chromosomes as the H3PS10 staining fades (Fig. 3B) suggesting that reassociation is coincident with dephosphorylation of H3 at the Ser10.

**Figure 4:**
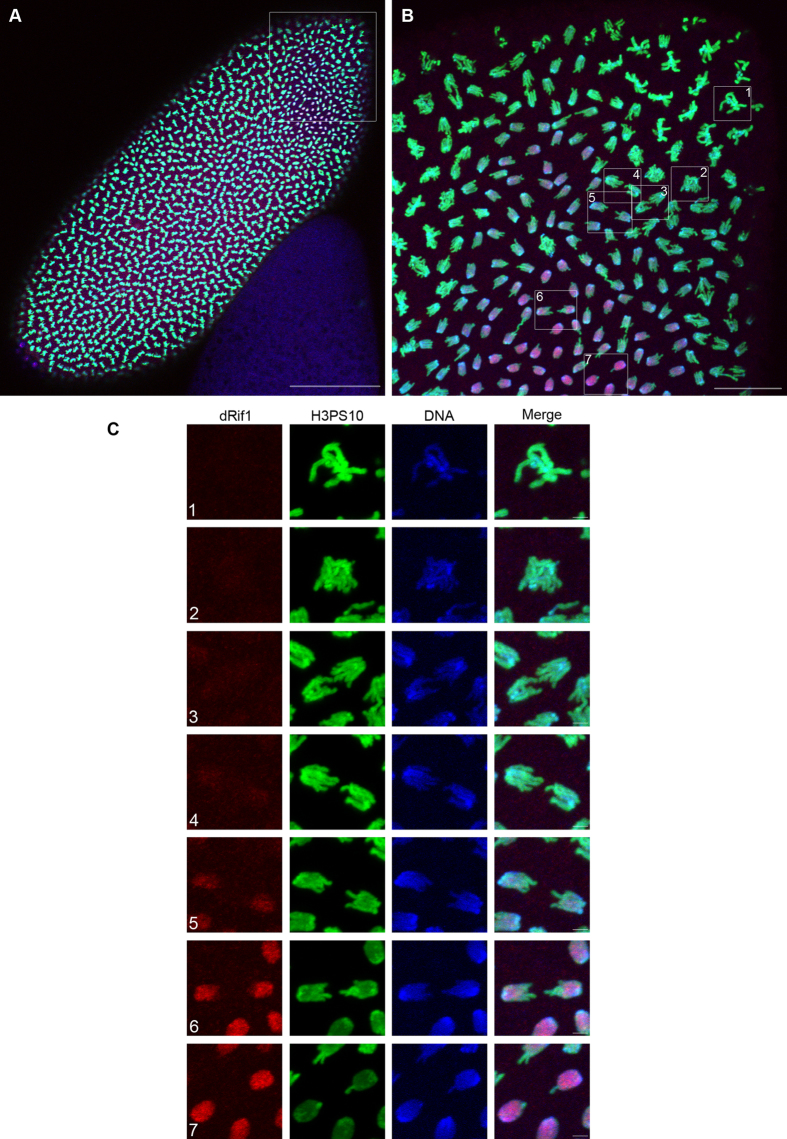
Dynamic association of dRif1 with mitotic chromosomes. *Drosophila* embryo stained with dRif1 (red) showing association to chromatin (TO-PRO3, blue) and this coincides with the diminishing of S10 phospho-specific H3-antibody (H3PS10, green).**A**) 20X image of the whole embryo just after losing synchrony in cell division. The scale bar represents 100 μm. **B**) The marked region of the embryo in the panel A imaged at 100X magnification. The scale bar represents 20 μm. **C**) Marked insets are magnified and arranged from metaphase (top) to telophase progression showing the re-association of dRif1 and dephosphorylation of H3PS10. Scale bar at 2 μm.

We further confirmed these observations with higher magnification imaging and observing single nuclei at different mitotic stages from the same embryo. As shown in Fig. 4, dRif1 (red) is absent in metaphase chromosomes and dRif1 re-associates with the condensed chromosomes during late anaphase which coincided with dephosphorylation of H3 at Ser10. The phospho-specific antibody staining (green) begins to diminish on the chromosomes as dRif1 signal increases, reinforcing that timing of dRif1 reassociation correlates with dephosphorylation of H3PS10. The inverse correlation of dRif1 association (red) and reduction of H3PS10 (green) is shown in the supplementary figure S3.

### dRif1 is essential for normal development of adults

In order to understand the function of dRif1 during development, we used UAS GAL4 based RNAi approach to study effects of its loss of function. We could not knock-down dRif1 completely in the embryonic stages using the RNAi lines generated in our lab or the lines obtained from VDRC even using strong and ubiquitous drivers like actin and tubulin. This was probably due to the very high levels of transcript in the early embryo. In these RNAi flies, however, dRif1 was knocked down to undetectable levels later in imaginal discs (Figure S4). This RNAi did not have any effect on development, and normal fertile flies emerged with this genotype. We could see no effect even after maintaining these flies in this context for several generations. We then induced UAS RNAi for dRif1 (VDRC*#* 33672) using *ActinGAL4* along with *UAS-Dcr2* (Bloomington*#*25708) to enhance RNAi effect. In this context, we could knock-down dRif1 to a greater extent, which resulted in lethality. Less than 20% (out of which 75% female and 25% male) flies do hatch, but only live for a few days, are infertile and females show increased necrotic spots in the abdominal segments. These observations establish that dRif1 is essential for normal development of adult flies.

### Ectopic expression of dRif1 leads to larval to pupal transformation defect and lethality

Since dRif1 shows a non-uniform expression pattern in later stages of development, we used ectopic expression to explore the effects of its gain of function. We used EP27427 line which has the EP element inserted in the 5’ UTR of dRif1 gene and can be induced by GAL4 drivers^24^. We confirmed expression of dRif1 in the larvae of these lines by western blots (Figure S6). Ubiquitous over expression of dRif1 in this line by *TubulinGAL4* or *ActinGAL4* lines leads to complete lethality. We found there was abnormality in larval to pupal transformation. Though all larvae made pupal cases, no adults emerged from them. Majority died as early pupae, a few progressed to late pupal stages. Removal of the pupal case revealed that most pupae died before organ formation, suggesting that the metamorphosis from larval to pupa was affected.

**Figure 5:**
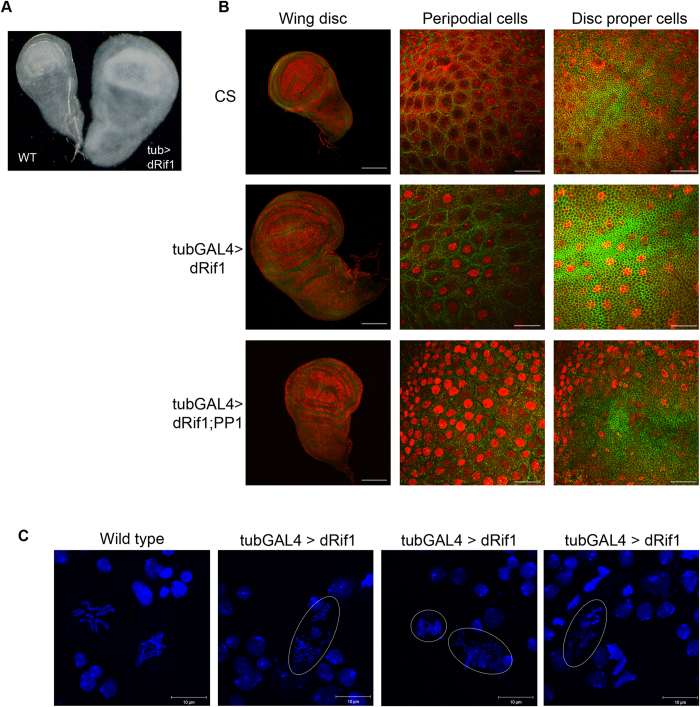
Phenotype of ectopic expression of dRif1. Ectopic expression of dRif1 was carried out using Tubulin-GAL4 driver and representative images shown here from 3^rd^ instar larval wing imaginal disc and neuroblast indicates defects in normal cell cycle progression and such larvae die as early pupa. **A**) Wing imaginal disc dissected from 3^rd^ instar larvae of wild type and larvae with ectopic expression of dRif1 captured side by side to compare size of these discs. **B**) Wing imaginal discs from indicated genotypes are shown along with scale bar. Co-staining of dRif1 (red) along with armadillo (green) in whole imaginal discs showing increased disc size and confocal sections at 100x magnifications of same discs showing peripodial cells and disc proper cells for cell size comparison. Both disc size and cell size is larger in ectopic dRif1 expression than CS flies (compare row 1 with row 2). Row 3: When dRif1 and PP1 87B were co-expressed, the imaginal disc size was like wild type and the cell size also reduced to the wild type level (compare row 3 with row 2 and row 1). The scale bar represents 100 μm in the lower magnification images and 20 μm in the higher magnification images. **C**) Chromosomes from neuroblasts isolated from 3^rd^ instar larvae of CS showing properly condensed chromosomes but condensation defects were observed (circled) in ectopic expression of dRif1 under *Tubulin*-*GAL4* driver. The scale bar represents 10 μm.

To discern the possible reason for this phenotype we dissected out the 3^rd^ instar larvae and observed the imaginal discs. We found a significant enlargement of the wing, eye, leg and haltere imaginal discs compared to the wild type disc (Fig. 5A, figure S5). Discs were generally about 50% larger than the wild type. Figure 5A shows a typical wing imaginal disc from a larva overexpressing dRif1 and a wild type side by side for comparison. We further examined if these enlarged discs were due to increase in cell number or increase in cell size. We performed this experiment by costaining dRif1 in wing imaginal discs with antibodies against armadillo which demarcates the cell margins (Fig. 5B). We found that while the numbers of cells in the wild type and overexpression lines were similar, the dRif1 over expression lines had much larger cells (Fig. 5B, row 2). Thus, overexpression of dRif1 leads to increased cell size in the imaginal discs.

### Ectopic expression of dRif1 induces chromosomal condensation defects in neuroblasts

As ectopic expression of dRif1 is detrimental to the larval to pupal transition, and in embryonic nuclei dRif1 showed dynamic association with chromosomes, we investigated if there were any chromrosomal defects in larvae ectopically expressing dRif1. As previous studies have shown that chromosomal defects are readily visualized in neuroblasts, we prepared neuroblasts from 3^rd^ instar larvae expressing dRif1 and examined chromosomes^25^,^26^,^27^. Neuroblast preparations were carried out as reported^27^ from *TubulinGAL4>* dRif1 3^rd^ instar larvae. Pulverized (Fig. 5C, panel 2&3) and improperly condensed (Fig. 5C, panel 3&4) chromosomes could be detected in these neuroblast cells, indicating chromosome condensation defects. The pulverised appearance is often due to the premature condensation in the S-phase leading to premature exit from S-phase resulting in under-replicated and over-condensed chromosome structures. Neuroblasts from *Canton*-S 3rd instar larvae were used as control, which did not have any prematurely condensed chromosomes (Fig. 5C, panel 1). These experiments show that ectopic expression of dRif1 interferes with normal chromosome condensation.

### Ectopic over expression of dRif1-phenotype can be rescued by over expression of PP1-87B

Several lines of observations suggest interactions between dRif1 and PP1. First, in our bioinformatic analysis, we had identified an evolutionarily conserved PP1 interaction motif, SILK-RVxF, in dRif1^22^. Second, genome-scale pull down experiments with tagged PP1-87B/dRif1 have shown that the two proteins interact^28^. Third, both the ectopic expression of dRif1 and the knock-down of PP1 cause early pupal lethality^29^,^30^,^31^. Fourth, depletion of PP1-87B was earlier reported to be defective in chromosome condensation^25^,^32^. More recently, interaction between Rif1 and PP1 has been demonstrated in yeast^19^,^20^,^21^.

**Figure 6:**
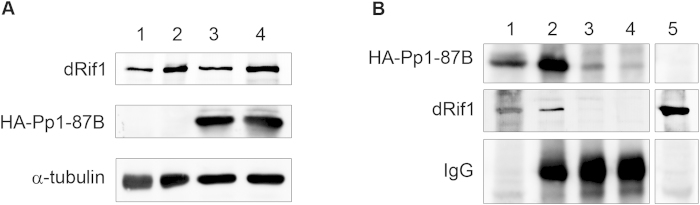
dRif1 interacts with PP1-87B. **A)** Western blot to detect expression levels of dRif1 and PP1 in WT (lane 1), showing increased expression of dRif1 in EP27427 (lane 2) ectopic expression of HA tagged PP1 87B (lane 3) and coexpression of dRif1 and PP1 87B together (lane 4). Same blots probed with tubulin for loading control. Uncropped images are shown in figure S6. **B)** Immunoprecipitation with HA (lane 2) or IgG (lane 3) was carried out from larval imaginal disc extracts expressing HA tagged PP1 87B under *TubulinGAL4* driver. HA pull down from extract with no HA-PP1expression used as control (lane 4). Total protein extract from larval imaginal discs were immunoprecipitated with antibodies to HA or control IgG and the immunoprecipitates were probed with anti-HA antibodies (row 1) and dRif1 antibodies (row 2). Row 3 shows the heavy chain of immunoglobulins that were used in the IP, indicating addition of antibodies to both the extracts. The input extract used for IP was loaded in lane 1(*TubulinGAL4* > HA PP1) and in lane 5 (no HA PP1 expression). dRif1 was detected only in pull downs with HA antibodies from *TubulinGAL4* > HA PP1 showing specific interaction with PP1. Lane 5 and lanes 1 to 4 in panel B are parts of the same blot and uncropped images are shown in figure S6.

We now tested if we could rescue the dRif1 ectopic expression phenotype by co-expressing PP1-87B. We simultaneously ectopically expressed both dRif1 and HA-PP1-87B under the *TubulinGAL4* driver. The additional expression of dRif1 and PP1 proteins were confirmed by western blotting (Fig. 6A). Wing imaginal disc extracts from 10 larval equivalents (10pairs) of wild type, *TubulinGAL4*>dRif1, *TubulinGAL4*> HA-PP1-87B and *TubulinGAL4*>dRif1; HA-PP1-87B were loaded and the blot showed increased expression of dRif1 in lanes 2, 4 and PP1 expression in lanes 3, 4. Co-expression of PP1 did not reduce level of dRif1 as seen in lane 4. We found that the lines co-expressing dRif1 and PP1-87B were viable and fertile showing that PP1 overexpression completely rescues the early pupal lethality associated with dRif1 overexpression. Further, staining wing imaginal disc of *TubulinGAL4*>dRif1; PP1-87B with antibodies against armadillo protein showed that their cell size was similar to those in wild type imaginal discs (Fig. 5B, row 3). These observations suggest that ectopic expression of dRif1 creates a shortage of PP1, that leads to increased cell size and lethality which can be rescued by providing additional quantities of PP1. The result of overexpression of dRif1 phenotype being rescued by coexpression of PP1 could be due to direct interaction of the two proteins or could be due to the convergence of divergent targets of the two.

In order to distinguish between the two possibilities, we experimentally tested if the two proteins interacted by coimmunoprecipitation experiments (Fig. 6B). Protein extracts were made from 3^rd^ instar larval imaginal discs in which the HA-PP1-87B is driven by *TubulinGAL4* driver and the pull down with HA-antibody was performed along with rabbit IgG control. Western blotting of the immunoprecipitates with dRif1 antibody detected endogenous dRif1 co-precipitating with PP1 in the HA pull down but not in the IgG control lane or in lines not containing HA tagged Pp1 suggesting that these two proteins interact physically. Uncropped imags are shown in figure S6.

### dRif1 functions in controlling timing of origin firing in *Drosophila* cells

**Figure 7:**
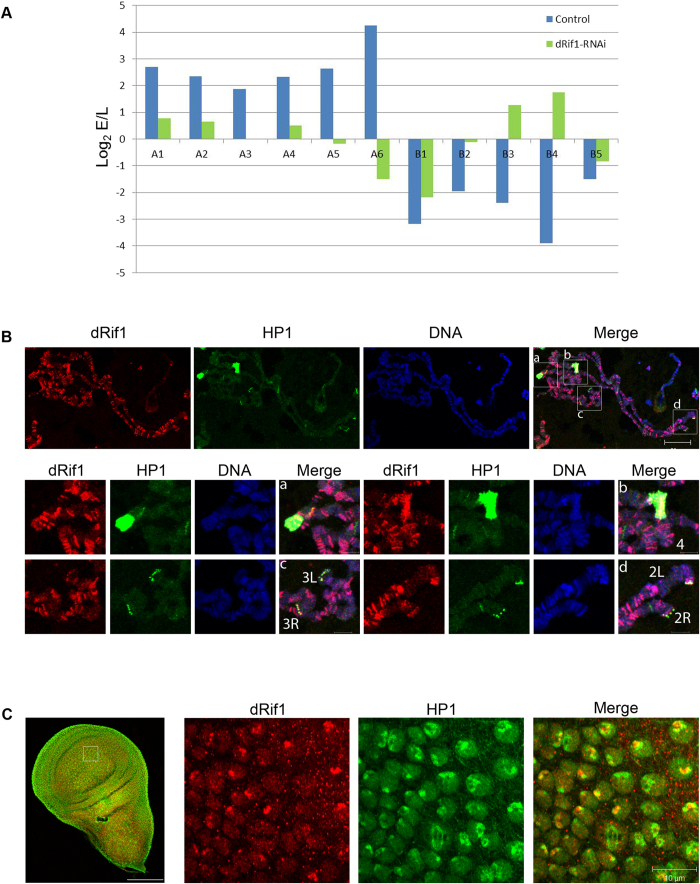
dRif1 regulates timing of replication initiation from origins and associates with HP1. **A)** S2 cells untreated (blue bar) or dRif1 RNAi treated (green bar) cells were arrested with HU and labeled with BrdU immediately for 1 hr early replication samples (E) or after 4 hours for late replicating samples (L). The y-axis represents log_2_E/L value, where log2 of the ratio of enrichment of early vs late for each locus is plotted. X-axis shows the selected euchromatic origins (A1-6) and heterochromatic origins (B1-5) from chromosome 2 and 3. A1-A2 and B1 –B3 are from 2L, A3-A4 and B4 are from 3L, A5-A6 and B5 are from 3R chromosomes. **B**) Polytene chromosome from the 3^rd^ instar larvae stained for dRif1 (red) and HP1 (green) showing dRif1 localization on multiple regions on polytene chromosomes. Magnified insets (–a-d) are heterochromatic regions (chromo-center and telomere) with intense HP1 foci and dRif1 co-localizes with HP1 in these regions. Arrow heads in the red panel shows that dRif1 localizes to the polytene telomere cytologically. The scale bar represents 20 μm in the polytene spread and 5 μm in the magnified insets. **C**) dRif1 (red) co-localizes with HP1 (green) in larval wing imaginal disc. The scale bar represents 100 μm in the lower magnification image and 10 μm in the higher magnification image.

Rif1 from both yeast and mammals has been shown to influence the timing of initiation of replication from origins of replication. Several lines of observations suggest that dRif1 may also be involved in this process. Our current studies in *Drosophila* embryos show that dRif1 reassociates with chromosomes in late anaphase/telophase and this timing coincides with the reassociation of pre-replication factors to chromatin^33^. In addition, there are several striking similarities between dRif1 and *Drosophila* Orc2 (DmORC2), mutants of which also show loss in temporal regulation of replication timing. DmOrc2 disassociates from chromosomes at the advent of metaphase and associates with late anaphase chromosomes and also colocalizes with HP1. Pulverized chromosomes and improperly condensed chromosomes that we observe with ectopic expression of dRif1 are also seen in DmORC2 mutants of *Drosophila*^27^. Considering these multiple indirect indicators, we tested if dRif1 was involved in controlling the timing of origin firing in *Drosophila* as well. In a recent genome-wide analysis of firing of replication origins, the timing of initiation of replication from all origins was mapped in *Drosophila* cell lines^34^. We had previously shown that dRif1 could be knocked down to undetectable levels in S2 cells, one of the embryonic cell lines whose origin firing landscape was documented. Therefore, using S2 cells, we tested if dRif1 had any role in the temporal regulation of origin firing. S2 cells were first subjected to RNAi of dRif1 (Figure S7) followed by immunoprecipitation of replication origins in both RNAi treated cells and normal, untreated cells. In order to track the origins of replication, normal and RNAi treated cells were first arrested with hydroxy urea (early S phase) and then allowed to reinitiate DNA synthesis in growth medium supplemented with BrdU to label the early origins or alternately, allowed to initiate replication in normal medium for 4 hours followed by addition of BrdU to label late firing origins (figure S8). This was followed by chromatin immunoprecipitation using antibodies specific to BrdU to enrich the origins of replication. Enrichment of origins of replication in the two sets of samples was quantified using quantitative Real time PCR. The PCR primers were designed based on the published data set describing the temporal landscape of origin firing in S2 cells. We tested several early and late origins from chromosome 2 and chromsosome 3. The data for a few representative origins is shown in Fig. 7A. We first confirmed that in normal cells, those origins described as early and late were indeed firing with the same temporal regulation and followed it up with estimating their timing of firing in RNAi treated cells. As shown in Fig. 7A, we find that these selected origins showed, as expected, early or late initiation of replication (blue bars). However in cells where dRif1 was knocked down, there was clear difference in initiation of replication from these origins. Several early origins were initiating late and many late origins were initiating early (green bars). We also find that in untreated cells each tested origin consistently initiated replication either early or late as expected. In the RNAi treated cells, there was variation between experiments for the same origin. However, in each case where dRif1 was knocked down, several origins were misregulated. The differences observed for individual origins between experiments may reflect the residual activity of dRif1 that remained in the RNAi treated cells or alternately, indicate random timing of initiation of replication due to loss in dRif1. These findings establish that dRif1 is important for temporal regulation of initiation of replication, making it the most conserved function of Rif1 from yeast to mammals.

### dRif1 associates with the telomere and co-localizes with HP1

Rif1 localizes predominantly to telomeres in yeast^4^ but not in mammalian cells^6^,^7^. In *Drosophila* cells, our earlier study found a strong association of dRif1 to heterochromatic regions in S2 cells^22^. Earlier studies in S2 cells showed that heterochromatin associated protein, HP1 is involved in modulating the replication timing in *Drosophila*^35^. In order to see whether dRif1 associates with HP1 in *Drosophila*, we performed colocalization experiments with dRif1 and HP1 in polytene chromosomes and imaginal discs. dRif1 localized to polytene chromosomes at many places (Fig. 7B), appeared to preferably localize to bands compared to interbands and importantly, it colocalized with HP1 at the chromocenter and telomeres (Pearson’s correlation coefficient of 0.5+/− 0.04; magnified insets; figure S8). Imaginal discs express dRif1 at low levels and about 60% of this also colocalizes with HP1 (Fig. 7C). These results suggest that a large fraction of cellular dRif1 is associated with heterochromatin in later stages of development and, unlike mammalian Rif1, it is also associated with unperturbed telomeres at least in the salivary gland polytene chromosomes in *Drosophila*.

## Discussion

Rif1, originally discovered as a negative regulator of telomerase activity in yeast, is an evolutionarily conserved protein. Although the telomere specific functions have so far not been demonstrated in other organisms except yeasts, Rif1 plays important roles in repair of DNA double strand breaks by regulating the choice of pathway for repair and recombination^9^,^10^,^11^,^12^,^13^ and in re-initiating replication from stalled forks^36^. In addition, in yeast, Rif1 also prevents the activation of DNA damage response at DNA breaks proximal to the telomeres^14^,^15^. A function of Rif1 conserved in both yeast and mammals is the temporal regulation of initiation of replication^16^,^17^,^18^. The molecular mechanistic basis for Rif1 function in any of these is not clear.

To investigate the molecular basis of Rif1 function in the organismal context, we used reverse genetic approach in *Drosophila*. We first analysed the expression profile of Rif1 and its developmental dynamics. dRif1 is abundant in early embryos and the amount diminishes as differentiation and organ formation progresses. Later in development, dRif1 is expressed in imaginal discs, in salivary glands and in the ovary. In most cells this protein colocalizes with HP1 including telomeres and chromocenter of polytene chromosomes. dRif1 also shows a dynamic association with chromosomes during mitosis; though present in the interphase and prophase nuclei, dRif1 is excluded from the condensed metaphase chromosomes only to reassociate with the late anaphase/early telophase chromosomes. dRif1-HP1 colocalization and the mitotic behaviour of dRif1 protein have implications for possible role in temporal regulation of replication initiation. Knock-down of HP1 in *Drosophila* cell line leads to loss in temporal regulation of replication initiation with multiple origins in the pericentromeric heterochromatin firing earlier and several origins near repetitive DNA firing later^35^. Like dRif1, Orc2, a component of the pre-replication complex, shows similar dynamic association with chromosomes during mitosis and also regulates the timing of replication initiation^27^,^33^. It is also known that HP1 and Orc2 interact with each other^37^. As these observations further substantiate a possible role for dRif1 in temporal regulation of replication in *Drosophila*, we investigated this possibility. Using RNAi approach to knock-down dRif1 in *Drosophila* cell line, S2, we show that temporal regulation of origin firing indeed requires dRif1 in *Drosophila.* Interestingly, among all the reported functions for Rif1 in various systems the temporal regulation of origin firing is conserved in yeast, *Drosophila* and human cells. Relationship between the timing of replication and expression state of genetic loci across the genome is well established^38^. Early firing of the origins in active regions of the genome and late firing of those in repressed regions has been implicated in the maintenance of differential state of expression at the genome level. While interfering with timing of firing of origins may not have serious consequences in cultured cells as seen in yeast or S2 cells, it is likely to have more impact at the organismal level. dRif1 is essential as knock-down leads to lethality at pupal stage. Escapers emerge as adults to survive only for a few days and are infertile. An essential role for dRif1 even during early development cannot be ruled out as a large amount of Rif1 is maternally deposited, and maybe sufficient to develop until pupal stages. It is also possible that temporal regulation of origin firing is more critical during late developmental stage of pupation. It is known that massive reprogramming and cell death is essential during transformation from third instar larvae for the formation of adult organs during pupation and maybe more sensitive to the loss in temporal regulation of replication initiation.

Our findings establish that dRif1 has a spatially restricted pattern of expression during late developmental stages and is essential for transition from larvae to pupa. We investigated the significance of the restricted pattern of expression of dRif1. Using the UAS-Gal4 system of inducible expression we show that ectopic or overexpression of dRif1 leads to early pupal lethality. dRif1 overexpressing larvae make pupal case but do not transform into pupae internally. We find that the imaginal discs in the dRif1 overexpressing larvae show remarkable increase in size. A closer examination revealed that the disc size increase is not due to the increased cell division and is instead due to increase in cell size. These observations in the context of chromosomal association of dRif1 suggest that ectopic or overexpression of dRif1 alters cell fate in such a way that the disc cells lose the potential to differentiate or divide and instead go on to increase cell size, typical of terminally differentiated cells. Since dRif1 shows dynamic association with chromosomes, its mis-expression could also affect the differentiation program by altering chromosome structure. This suggestion is supported by our observation of improper chromosome condensation and pulverized chromosomes in neuroblasts. These findings indicate that dosage of dRif1 protein is of critical importance for normal development in *Drosophila*. Interesting parallels between dRif1 and Rif1 in mammals include abundant expression in early development and low levels of expression in later stages of development in both systems; requirement for Rif1 in embryonic stem cells in mammals and in larval to pupal transition in flies when organogenesis takes place and finally, involvement of Rif1 in telomere position effect in mammals and in *Drosophila,* a close association with HP1, a protein involved in telomere position effect and replication timing control. It would be of interest to study if these links are a manifestation of an underlying conserved function of Rif1 in the two systems^8^,^35^,^39^,^40^.

**Figure 8:**
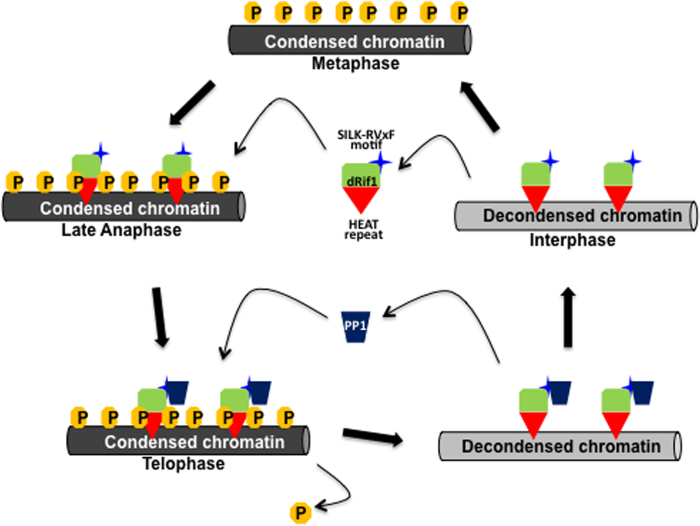
A model depicting the proposed action of Rif1. In interphase cells, Rif1 is associated with chromosomes. When chromosome condensation takes place at the onset of mitosis, Rif1 dissociates from chromosomes and reassociates at the late anaphase stage. We propose that at this stage it interacts with PP1 via the SILK-RVxF motif and recruits PP1 to the chromosomes. This recruitment leads to dephosphorylation of substrates on the chromosome and decondensation of chromosomes. PP1 and Rif1 probably disassociate at this stage. The association and dissociation of Rif1 from chromosomes may be mediated by modifications of Rif1protein.

Having established a key role for dRif1 in the developmental program of *Drosophila* we looked for the molecular basis of its function. One of the ways that dosage of a protein may affect development is by disturbing the interacting partners; either by sequestering interacting partners in non-functional complexes or by targeting them to ectopic sites that are not normally targeted. While the known Rif1 partners like Rap1, Rif2, 53BP1 have not been reported in *Drosophila,* in our earlier study we had identified a SILK-RVxF motif that is known to interact with PP1. Earlier pull down experiments^41^ and recent reports in yeast^19^,^20^,^21^ confirm this. Here we demonstrate that dRif1 and PP1 interact physically in *Drosophila*. Most striking is our observation of complete reversal of the lethality phenotype of dRif1 ectopic expression by parallel, induced expression of PP1 in these lines. PP1 expression rescues both the lethality and the abnormal growth of imaginal discs where the cell size returns to normal. This implicates PP1 as the key effector for dRif1, and suggests that dRif1 could be targeting PP1 to specific genomic sites. Strong support for dRif1 to function as adapter for PP1 to target specific sites in the nucleus comes from several observations. The localization of dRif1 to chromosomes in telophase coincides with the dephosphorylation of histone H3S10 phosphorylation, an activity that has been attributed to PP1^42^. Even though many protein kinases and their specific substrates have been identified, substrate specificity of phosphatases is poorly understood. Protein phosphatase 1 family of proteins has diverse functions and the catalytic subunits show no substrate specificity. *In vivo*, specificity is conferred by the so-called adaptor proteins that recruit phosphatases to specific sites. We propose that dRif1 could act by recruiting PP1 to origins of replication and other genomic loci where dephosphorylation of associated chromatin factors may be needed. However, we could not detect dRif1 at the origin of replication regions which were tested in this study by chromatin immunoprecipitation. A similar result was obtained in yeast, where Rif1 could be detected only at origins in the sub-telomeric region^19^. HEAT repeat has been proposed to be associated with chromatin associated proteins earlier^43^ and we had identified a Rif1 specific HEAT repeat region^22^. We predict that dRif1 associates with chromosomes through HEAT repeats and recruits PP1 via the SILK-RVxF motif and also perhaps other proteins to the chromosomes. A model for dRif1 function based on our results is depicted in fig. 8. In dividing cells, Rif1 is associated with the chromosomes. In mitosis at the metaphase stage, Rif1 leaves the chromosomes and in late anaphase, prior to dephosphorylation of histones, it reassociates with chromsomes. We predict at this stage it recruits PP1 to chromosomes leading to dephosphorylation of histones and possibly other substrates including pre-replication factors that reassociate with the origins at this stage. This dynamic association has been shown for mammalian Rif1 as well and PP1 interactions are also conserved across evolution. Thus this model may be universally applicable. It would be intersting to understand the cause for this dynamic association of Rif1 with chromosomes.

Since PP1 interacting SILK-RVxF motif of Rif1 is conserved in yeast, fly and mammals, it is likely that the Rif1-PP1 interaction demonstrated by us in this study and by others in yeasts is also the case for the mammalian systems. The *in vivo* functional significance of this interaction is clearly demonstrated by our finding of phenotypic suppression and rescue of dRif1 ectopic expression by PP1 co-expression. Considering that regulation of replication timing is the only common function of Rif1 seen in yeast, fly and human, it is likely that temporal regulation by Rif1 is executed by recruiting PP1 to the origin of replication and that this mechanism is conserved during evolution. Recent work from yeasts supports this suggestion^19^,^20^,^21^. In addition, it is possible Rif1 may also recruit PP1 to other sites where it is proposed to function namely, to telomeres in yeast and to DNA damage sites in mammals.

Our study introduces a new model system to investigate the function of Rif1 at the organismal level and in the context of development. We also provide evidence for one new evolutionarily conserved interactor for Rif1, which in turn, provides one key mechanism through which Rif1 may function at multiple locations. Furthermore, this work lays the ground for further studies to interrogate the mechanisms of functions of Rif1 evolutionarily conserved in general and consequences of altering the temporal regulation of origin firing in particular.

## Methods

### Drosophila experiments

Canton-S flies were used as wild type in all experiments. For generation of flies ectopically expressing dRif1, the EP-line inserted into the 5’-UTR of dRif1 (Bloomington No. 27427; P{EP}*Rif1*[G18022]) was used. EP-line was crossed to *Tubulin-GAL4/TM3, Ser.GFP*, to identify larvae ectopically expressing dRif1 based on the absence of GFP-balancer. To identify the stage of lethality, we separated larvae based on GFP and kept 50 larvae of each type in different vials and observed the phenotype. GFP containing larvae served as control. To rescue the lethality associated with the ectopic expression of dRif1, HA tagged PP1-87B (Bloomington No. 24098; w[*]; P{w[+mC]=*UAS-Pp1-87B.HA*}3/*TM6C*, cu[1] Sb[1]) was brought to the EP-line on the III chromosome balanced over TM6B, Tb. This fly was crossed to *Tubulin-GAL4/TM3, Ser.GFP* to drive dRif1 and PP1-87B together under *Tubulin-GAL4*. For knocking down dRif1, VDRC line (VDRC No. 33672, homozygous on II chromosome) were crossed to *Actin5C–GAL4* flies which also has *UAS-Dcr-2.D* on the I-chromosome (Bloomington No. 25708; *P{w[+mC]=UAS-Dcr-2.D}1, w[1118]; P{w[+mC]=Act5C-GAL4*}25FO1/CyO). Crosses were set and maintained at 25 °C.

### RNA and protein methods

Total RNA from each sample was isolated using TRIzol (Invitrogen) reagent, separated on denaturing agarose gel, transferred to positively charged nylon membranes (IMMOBILON-NY+, Millipore) and probed with full length dRif1 using standard methods. Total protein was isolated from tissues or from different developmental stages by homogenizing them in SDS loading buffer and heating at 95 °C for 2-3 mins. For IP, total protein was isolated from imaginal discs from 3rd instar larvae of *tubulin Gal4*>*PP1-87B* genotype in 1X RIPA buffer containing 1X protease inhibitor cocktail.

### Embryo collection and Immunofluorescence

To stage the embryos, flies were kept in embryo collection cages and the embryos laid on fly food plates were collected. The plates were kept overnight, changed again and the first 2 hr collection discarded and next 2 hr collection was taken and labeled as 0-2 hr. Several 0-2 hr collection plates were staged by incubating for 2 hr, 4 hr etc. to obtain 2-4 hr, 4-6 hr up until 16-18 hr at 25 °C. Embryos were dechorionated, fixed in formaldehyde, de-vitellinized using methanol and stained with antibodies at appropriate dilutions. The embryos were blocked for 1 hr in 1XPBS + 0.1%TritonX100 + 0.1%BSA (PBST-BSA) followed by primary antibody incubation (overnight, 4 °C), washing, secondary antibody incubations (3 hrs, RT) and final wash. Washing was performed as 3 quick washes followed by 3 washes for 20 mins each, all in PBST and embryos were mounted in DAPI containing mounting media from Vectashield. Images were taken in Zeiss LSM-510 Meta confocal microscope, processed and exported using LSM software. Final figures were made in Adobe Photoshop. For staining imaginal discs, third instar larvae were dissected in PBS and fixed in 4% paraformaldehyde for 20 minutes. Blocking was for 1 hour in PBST-BSA followed by primary antibody incubation (overnight, 4 °C), washing, secondary antibody incubation (1 hr, RT) and final wash. Washing was performed three times with PBST for 15 minutes each. Discs were mounted with mounting media containing DAPI or TOPRO-3. For staining ovaries, ovary was dissected from female flies (fed with yeast for 2 days) in 1X PBS, fixed in PBST with 4% formaldehyde for 20 minutes, washed 3-4 times and blocked in PBST-BSA for 1 hr. This was followed by incubation with primary antibody, washing, secondary antibody incubation and final wash. Washes were 4 times for 1 minute each and once for 15 minutes with PBST. After washing, ovaries were spread in mounting media containing DAPI on a slide and sealed with a coverslip. dRif1 antibody was described previously^22^. Antibodies to H3PS10, PCNA, tubulin and HA tag were procured from Abcam and lamin-Dm0, HP1 and armadillo from DSHB. Antibodies were used as per recommended instructions. Fluorescence conjugated secondary antibodies, anti-rabbitCY3, anti-mouseCY3, anti-rabbit488, anti-mouse488, anti-rat488 and anti-mouseCY5 were obtained from Jackson Immuno Research.

### Neuroblast preparation

*Tubulin-GAL4/TM3-Ser-GFP* virgin flies were crossed to EP27427 male flies and collected non-driver (non-GFP) containing 3^rd^ instar larvae, which express dRif1 driven by Tubulin-GAL4. CNS was dissected from these 3^rd^ instar larvae and neuroblast preparation was carried out as described previously^27^.

### Replication timing and BrdU immunoprecipitation

ChIP was carried out as described^34^ using Brdu antibody (Life Technologies). Briefly, mock treated or dRif1-RNAi cells were treated with 1 mM hydroxy urea (HU) for 3 hrs, thereafter to one set 50 μg/ml BrdU was added and incubated for 21 hrs. The other set was incubated without BrdU for 21 hrs. Following this, both the sets were washed twice with ice cold 1XPBS to remove HU. To the first set BrdU was added for an additional 1 hr and harvested after that and used as ‘Early S phase sample’. The second set was incubated for additional 4 hrs to allow S phase progression and then BrdU was added and incubated for 2 hrs, harvested and used as ‘Late S phase sample’. DNA was isolated from these samples, sheared to get fragments enriched for less than 1 kb and BrdU IP was performed along with parallel IgG controls. Real time PCR was performed with experimental triplicates and BrdU enrichment was calculated by using percent input method. The percent of BrdU immunoprecipitated out of total incorporated was calculated for both early and late samples, the log ratios of Early/Late enrichment was plotted against indicated loci. Early and Late origin of replication loci were chosen based on the available modENCODE data. Early origin loci were taken from the euchromatic region which showed high peak in not only in S2 cells but also in other cell lines (Kc167 and Bg3). These loci did not show peaks in late S phase and were high in the early S phase in the RepliSeq track as well. All the regions chosen also were enriched for MCM and ORC binding indicating they are authentic replication origins. For late replication loci, regions were chosen from the heterochromatic regions showing no peak in the early S phase but showing high peak in the late S phase in the Repliseq.

## Additional Information

**How to cite this article**: Sreesankar, E. *et al. Drosophila* Rif1 is an essential gene and controls late developmental events by direct interaction with PP1-87B. *Sci. Rep.* **5**, 10679; doi: 10.1038/srep10679 (2015).

## Supplementary Material

Supporting Information Supplementary Figures 1-3 and Supplementary Tables 1-3
