# Corrigendum: *Drosophila* Rif1 is an essential gene and controls late developmental events by direct interaction with PP1-87B

**DOI:** 10.1038/srep17126

**Published:** 2016-02-09

**Authors:** Easwaran Sreesankar, Vellaichamy Bharathi, Rakesh K. Mishra, Krishnaveni Mishra

Scientific Reports 5: Article number: 1067910.1038/srep10679; published online: 05292015; updated: 02092016

In the Supplementary Information file originally published with this Article, Figures S3 and Figure S8 were omitted. In addition, Figures S4, S5, S6 and S7 were incorrectly labelled as Figures S3, S4, S5 and S6 respectively. These errors have now been corrected in the Supplementary Information that now accompanies the Article.

