# Supplementary material for: Drosophila Rif1 is an essential gene and controls late developmental events by direct interaction with PP1-87B
Source: Sci Rep. 2015 May 29;5:10679. doi: 10.1038/srep10679 (PMC4448129; doi:10.1038/srep10679)
Supplement: Supporting Information — Supplementary Figures 1-3 and Supplementary Tables 1-3 [file srep10679-s1.pdf]

Supplementary figures

Drosophila Rif1 is an essential gene and controls late developmental events by direct interaction with PP1-87B

Easwaran Sreesankar, Vellaichamy Bharathi, Rakesh K. Mishra and Krishnaveni Mishra

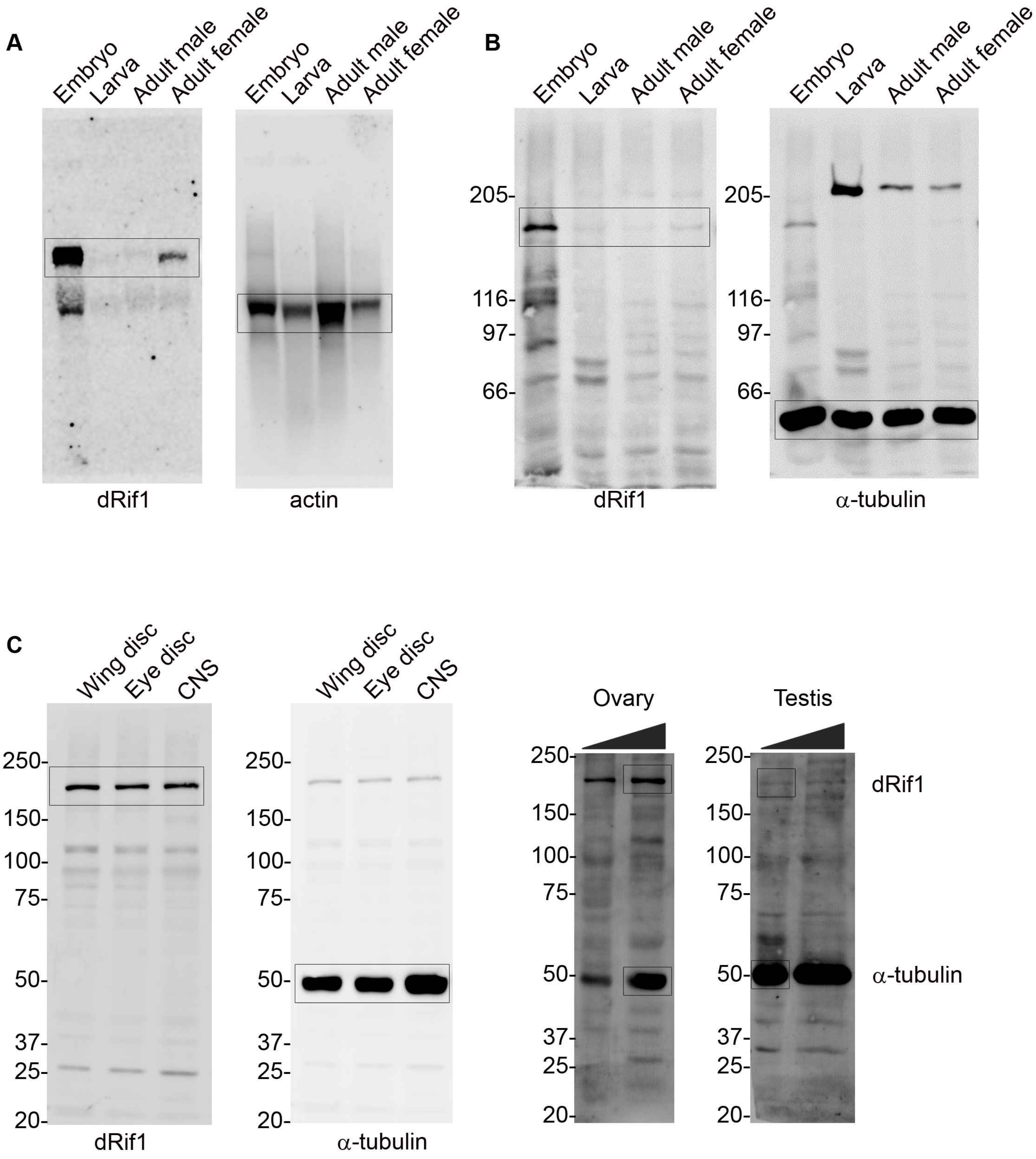

Figure S1: Expression of dRif1 in different developmental stages. Northern (A) and western (B-C) blots from figure 1 are shown as full length blots depicting the cropped area. All the control blots are carried out onto the same blot by using control probe/antibody.

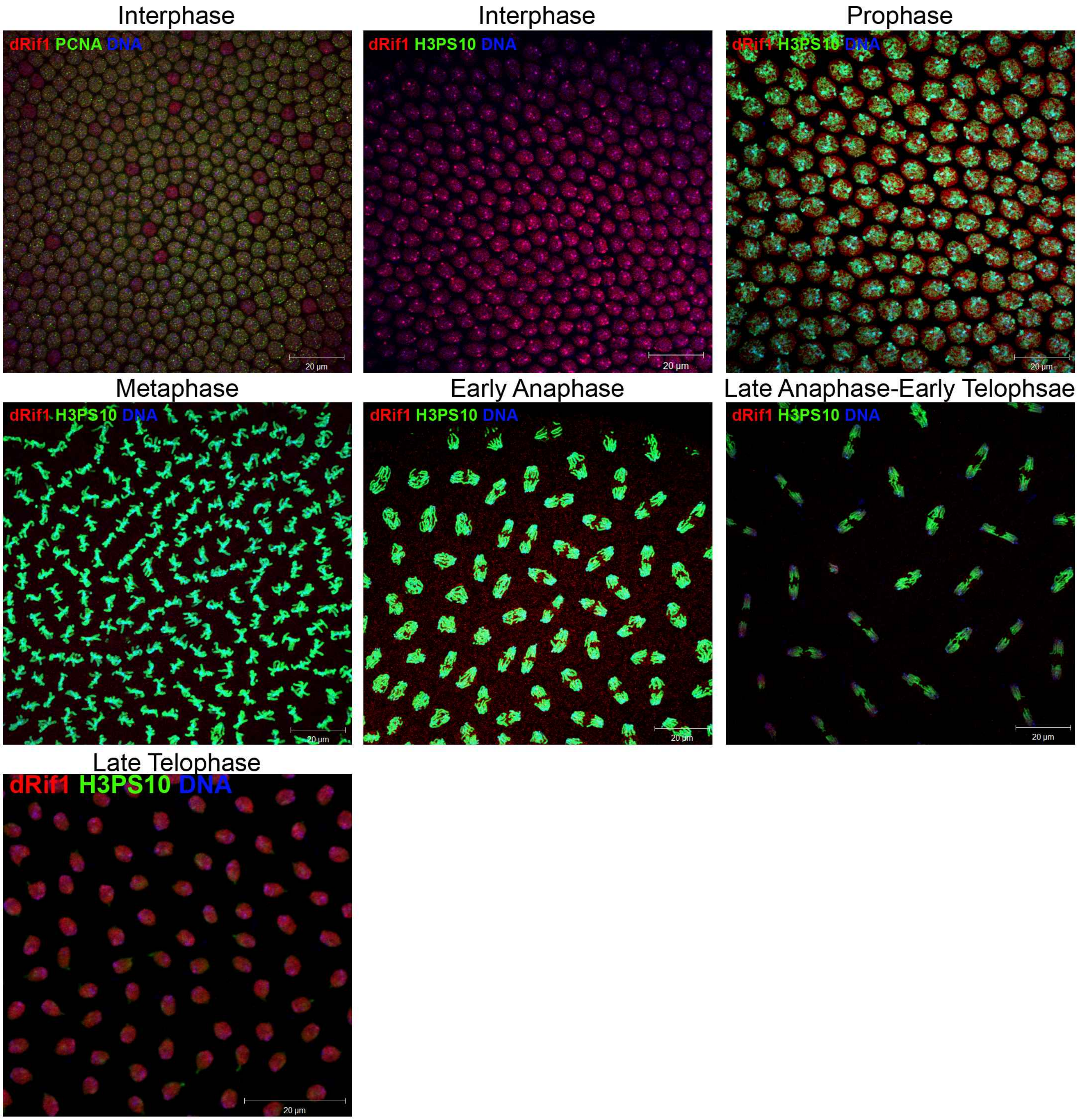

Figure S2: dRif1 localization in various stages of cell cycle in early embryo. Whole embryos were stained with anti-dRif1 antibody (red) along with G1-S phase marker, PCNA (green) or mitotic marker, H3PS10 (green) and DNA is stained with TOPRO-3 (blue). Interphase cells show large amounts of dRif1 (panel 1, 2) many nuclei in S phase show PCNA (panel 1) and do not contain H3PS10 (panel 2). Chromosomes condense in prophase and dRif1 can still be detected (panel 3), metaphase and early anaphase chromosomes are devoid of dRif1 (panels 4, 5) and as cells enter late anaphase/early telophase, dRif1 accumulates and by end of telophase chromosomes can be costained with dRif1. The scale bar represents 20µm.

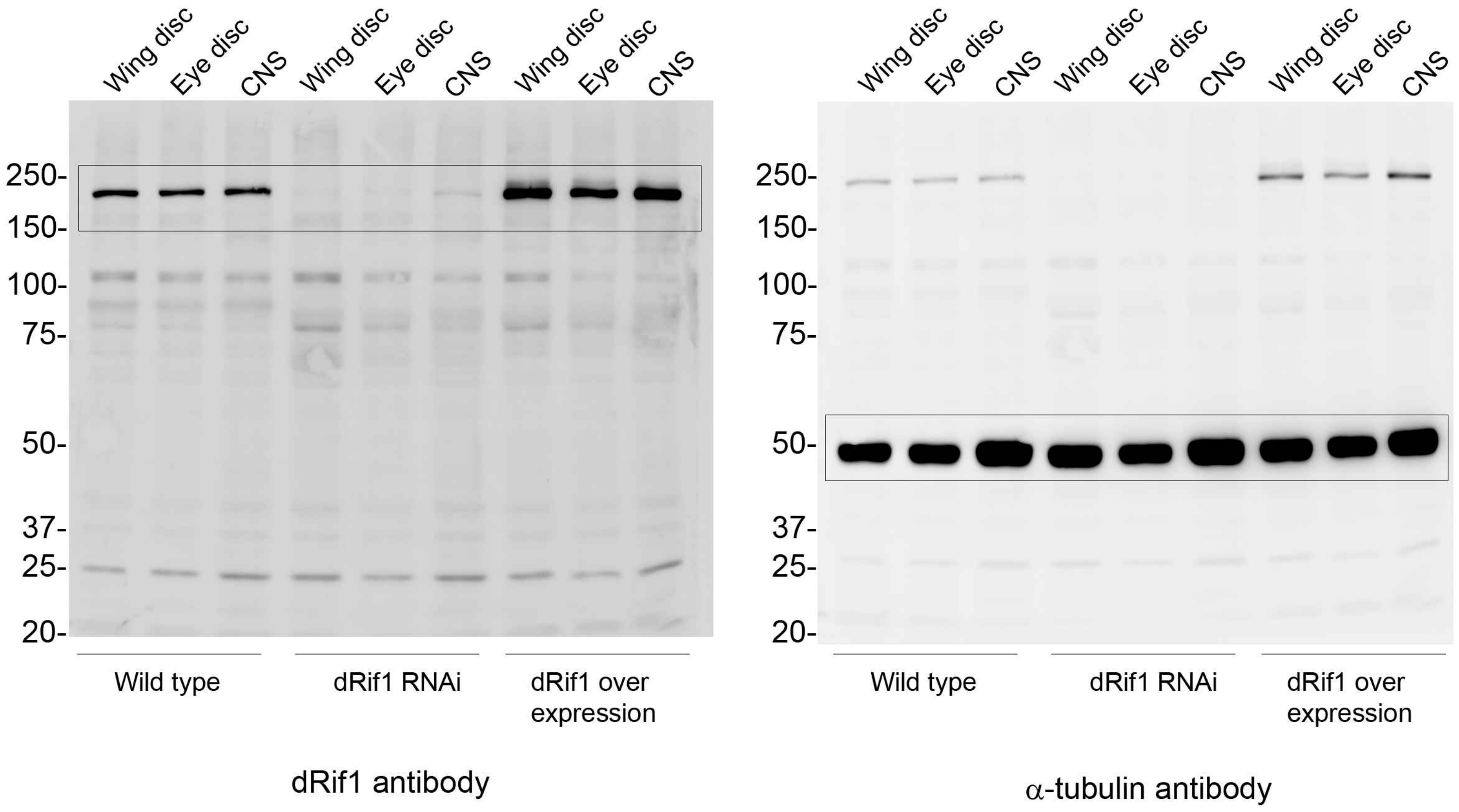

Figure S3: dRif1 RNAi and ectopic expression of dRif1 in larval imaginal discs. Total protein extracts were tested for dRif1 protein levels in wild type (lane 1-3), VDRC 33672 line driven using tubGAL4 driver to induce RNAi (4-6). Protein was almost undetectable in 3rd instar imaginal discs and in CNS a small amount could be detected. Lanes 7-9 show ectopic expression in EP27427 line driven using tubGAL4 driver; increased levels of dRif1 could be seen. Same blots were probed with antibodies to tubulin as loading control.

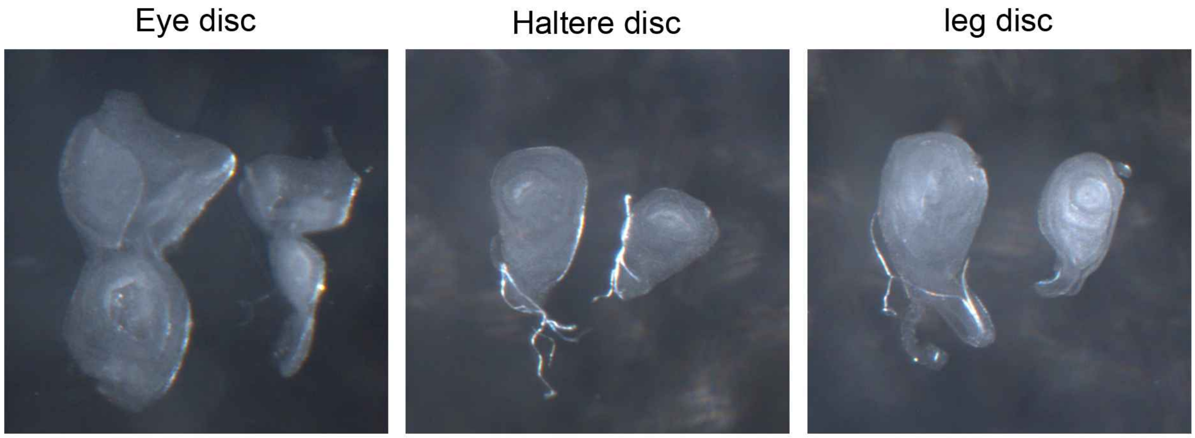

Figure S4: Ectopic expression of dRif1 leads to increase in imaginal disc size. Imaginal discs were dissected from 3rd instar larvae of either tubGAL4>dRif1(left) or wild type control (right) larvae were imaged side by side to compare the size. Eye, haltere and leg imaginal discs are shown here from a representative set as labeled.

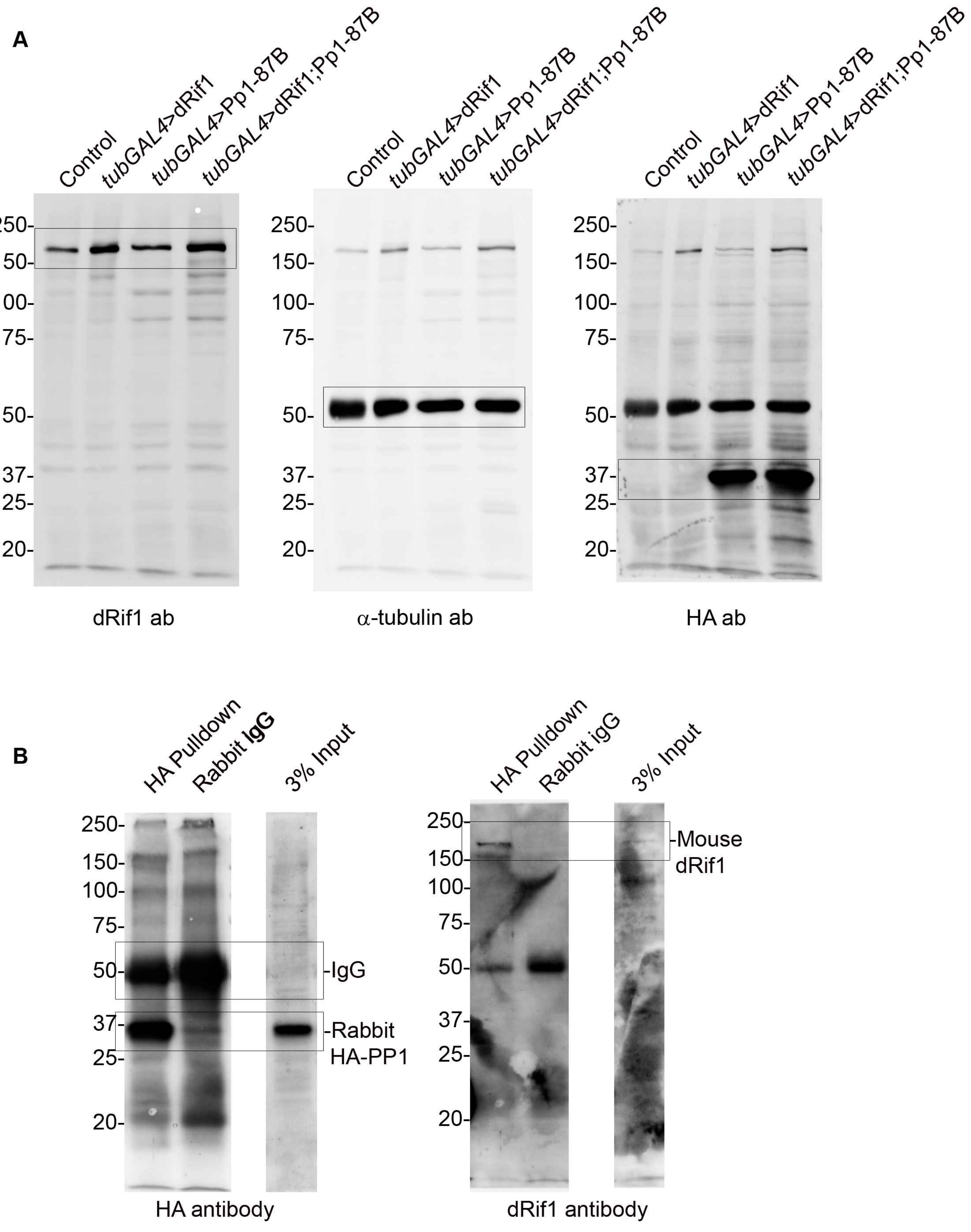

Figure S5: dRif1 interacts with PP1-87B. Full length blots from figure 6 are shown along with the cropped area marked. Same blots were probed with different antibodies as depicted in the images. The blots in panel B are parts from the same blot.

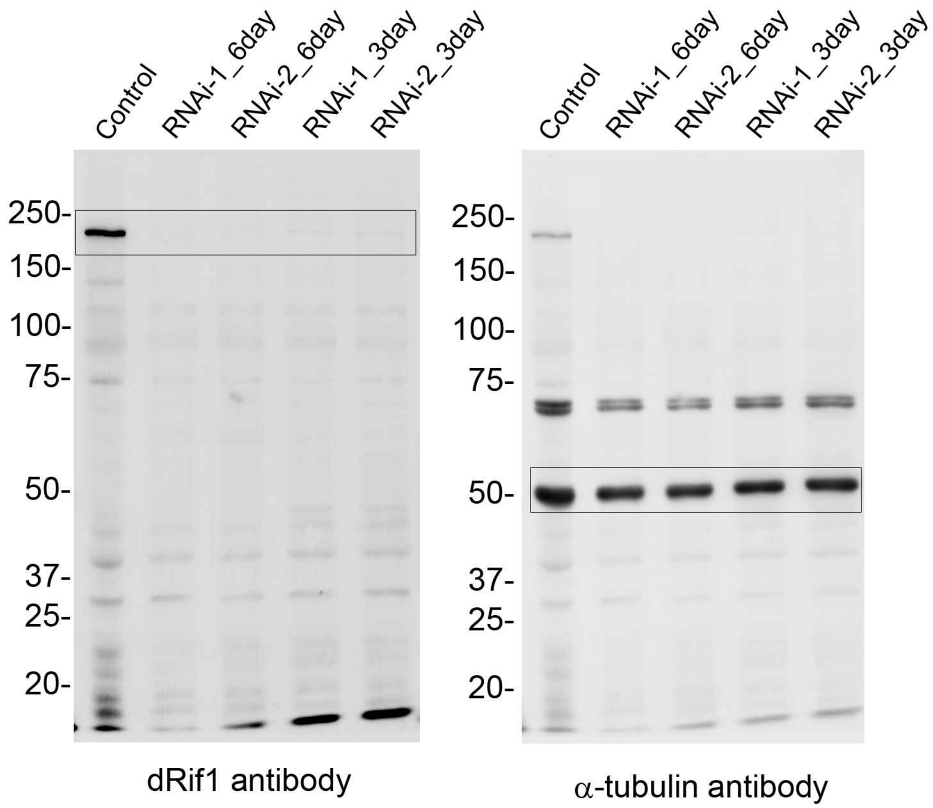

Figure S6: dsRNAi induced knockdown of dRif1 in S2 cells. Western blot with dRif1 of total protein extracts from S2 cells were done for untreated (lane 1), 3rd day after initial dsRNA treatment (lane 4, 5) followed by 3 days after second round of dsRNA treatment (lane 2, 3). 2nd round of dsRNA treatment which reduced the protein level considerably as seen from the 6th day protein extracts, was used for the experiments.
